# Changes in biochemical analytes in female dogs with subclinical *Ancylostoma* spp. infection

**DOI:** 10.1186/s12917-016-0833-2

**Published:** 2016-09-13

**Authors:** Elizabeth M. S. Schmidt, Asta Tvarijonaviciute, Silvia Martinez-Subiela, José J. Cerón, Peter D. Eckersall

**Affiliations:** 1 Department of Veterinary Clinical Sciences, School of Veterinary Medicine and Animal Science, São Paulo State University (FMVZ -UNESP), Campus Botucatu. Distrito de Rubião Jr, s/n. 18.618-000, Botucatu, SP Brazil; 2 Interdisciplinary Laboratory of Clinical Pathology, Interlab-UMU, Campus of Excellence Mare Nostrum, University of Murcia, Murcia, Spain; 3 Institute of Biodiversity, Animal Health and Comparative Medicine, College of Medical, Veterinary and Life Sciences, University of Glasgow, Glasgow, UK

**Keywords:** Acute phase proteins, C-reactive protein, Haptoglobin, Hookworm, IGF-1, Iron

## Abstract

**Background:**

*Ancylostoma* spp. is one of the most prevalent canine intestinal nematode infections which usually causes subclinical disease in adult dogs and has zoonotic implications. Therefore, the aim of this study was to explore and evaluate the possible pathophysiological changes that *Ancylostoma* spp. could produce in female dogs naturally infected but without clinical signs of disease, by screening a wide variety of biochemical markers for potential changes. Samples of feces and blood of 45 dogs were collected and fecal flotation and zinc sulphate centrifugal flotation were performed. The biochemical analytes determined were: the acute-phase proteins C-reactive protein (CRP) and haptoglobin (Hp); the lipid profile (cholesterol, triglycerides, HDL, LDL); the serum iron profile: iron, unsaturated iron binding-capacity (UIBC), and ferritin; the enzyme butyrylcholinesterase (BChe); the pancreatic profile: amylase, lipase, and trypsin-like immunoreactivity (TLI); the oxidative stress markers: total antioxidant capacity (TAC) and paraoxonase −1 (PON-1), along with total protein, albumin, and insulin-like growth factor – 1 (IGF – 1). *Ancylostoma* spp. eggs were detected in 29/45 dogs (64.4 %). Dogs were divided into two groups according to the results of fecal flotation methods. Group 1: negative fecal floatation (*n* = 16), and Group 2: subclinical infection with the observation of *Ancylostoma* spp. type eggs/x 40 objective fields (*n* = 29).

**Results:**

Mann–Whitney *U* test was used to compare the biochemical analyte results between the two groups (*P* < 0.05). Significant increases in CRP (μg/mL) (median): non-infected dogs: 5.5; subclinically infected dogs 18.7; *P* = 0.03, Hp (g/L) (median): G1: 2.4; G2: 3.3; *P* = 0.03, and UIBC (μg/dL) (median): non-infected dogs: 139.4; subclinically infected dogs: 216; *P* = 0.0015, and significantly decreased iron (μg/dL) (median): non-infected dogs: 202.5; subclinically infected dogs: 125.7; *P* = 0.0041, IGF-1 (ng/mL) (median): non-infected dogs: 224; subclinically infected dogs: 123; *P* = 0.02, and albumin (g/dL) (median): non-infected dogs: 2.8; subclinically infected dogs: 2.5; *P* = 0.04 concentrations were observed in dogs with subclinical *Ancylostoma* spp. infection when compared to non-infected dogs.

**Conclusion:**

These findings provide an overview of the biochemical effects produced by patent *Ancylostoma* spp. in naturally infected dogs without any evident clinical signs of disease, which could be considered in differential diagnosis, especially in an endemic area for this parasite.

## Background

The hookworm *Ancylostoma caninum*, *Ancylostoma braziliense,* and *Ancylostoma ceylanicum* are among the most prevalent canine helminths. Infection is potentially fatal in young dogs and subclinical in adult dogs. It has zoonotic potential [1, 2], as *A. braziliense*, *A. caninum,* and *A. ceylanicum* are etiologic agents of human cutaneous larva migrans, eosinophilic enterititis, anemia, and diarrhea [2–5].

In Brazil, the genus *Ancylostoma* is the most frequently detected nematode infection in adult dogs and *A. caninum* is spread throughout the country, presenting serious public health implications due to its zoonotic potential [6, 7]. Dog feces, shed in crowded cities, contaminate the environment with parasite eggs and larvae, thereby contributing to zoonotic transmission. In southern regions of Brazil, São Paulo and Curitiba, high soil *Ancylostoma* spp. contamination has been recorded (46.8 % and 40.3 %, respectively) [8–10].

*Ancylostoma* spp. infects the host mainly through skin penetration or by the ingestion of the third larvae stage which directly reaches the small intestine [11]. In dogs *Ancylostoma* spp. infection typically results in diarrhea, blood loss and anemia, poor weight gain, dehydration and can affect dogs at any age [12, 13]. When adult dogs are infected, some larvae invade different body tissues, such as the gastrointestinal tract and skeletal muscles and enter an arrested development phase, resilient to most chemotherapeutic agents. In bitches they can be reactivated during estrus and in the terminal 2 to 3 weeks of pregnancy. They may then be passed via the milk to the litter, for at least one week after parturition [1, 10, 11, 14]. Fourth-stage larvae and adult worms burrow deeply and massively into the small intestinal mucosa. The parasites are active blood-feeders when attached, by the teeth in their globular buccal capsules, at the intestinal mucosa and thus symptoms might be severe and life threatening especially in puppies in which the peracute and acute disease and blood loss is potentially fatal [5, 11]. Intestinal infection may occur in female adult dogs and due to age-associated immunity the parasite usually causes subclinical infection in these cases, with eggs shedding in feces but without clinical signs [12, 13].

We hypothesized that adult dogs infected with *Ancylostoma* spp. but without clinical signs could have pathophysiological changes involving inflammation, protein, lipid and iron metabolism, pancreatic function, and oxidative stress. Therefore, the aim of this study was to explore and evaluate the possible pathophysiological changes that *Ancylostoma* spp. could produce in female dogs naturally infected but without clinical signs. For this purposes a wide variety of biochemical markers were screened for potential changes, including the concentrations of selected acute phase proteins (APPs) to evaluate inflammation such as C-reactive protein (CRP), haptoglobin (Hp), IGF-1, and albumin; a profile to evaluate lipid metabolism [cholesterol, triglycerides, HDL, LDL, and butyrylcholinesterase (BChe)], a profile to evaluate iron metabolism [iron (Fe), unsaturated iron binding-capacity (UIBC), and ferritin], a pancreatic profile: amylase, lipase, and trypsin-like immunoreactivity (TLI); and oxidative stress markers such as total antioxidant capacity (TAC) and paraoxonase −1 (PON-1).

## Results

*Ancylostoma* spp. eggs were detected in 29/45 dogs (64.4 %). Dogs of the both groups were apparently healthy at clinical examination, not showing clinical signs of cachexia, weight loss or diarrhea.

Biochemistry analytes in healthy and infected dogs are presented in Table 1. Statistically significantly (*P < 0.05*) higher concentrations of CRP (3.2 fold), Hp (1.4 fold) and UIBC (1.5 fold) were observed in infected dogs when comparing with non-infected dogs. In contrast, significantly (*P < 0.05*) lower levels of iron (1.4 fold), IGF-1 (1.6 fold) and albumin (1.1 fold) were observed in infected dogs when compared to non-infected dogs. Fold values represent fold differences between the median values of each group.

**Table 1:** Results of the comparison of CRP, Haptoglobin, and biochemical analytes between dogs non-infected and dogs subclinically infected with *Ancylostoma* spp.

Analytes	Non infected Median [range]	Subclinically infected Median [range]	U value	*P* value	Reference range
CRP (μg/mL)^a^	5.5 [5.0–8.7]	18.7 [5.0–82.7]	142	0.03	<12
Haptoglobin (g/L)^ab^	2.4 [1.1–3.7]	3.3 [1.4–5.5]	66	0.03	<3
IGF-1 (ng/mL)^ab^	224 [63–541]	123 [6–123]	66	0.02	50–400
TLI (μg/L)	9.9 [2.6–14.3]	7.8 [2.1–36.8]	66	0.59	5.4–32
Iron (μg/dL)^ab^	202.5 [99.5–437.4]	125.7 [13.0–443.0]	113	0.0041	81–220
UIBC (μg/dL)^a^	139.4 [18.8–268.5]	216 [5–332]	97.5	0.0015	130–300
Ferritin (μg/L)	107.3 [15.6–197.2]	121.3 [45.1–721.4]	205.5	0.53	60–190
BChe (mmol/mL)	2.6 [1.6–4.5]	2.5 [0.4–5.0]	201	0.46	3–5
PON-1 (UI/mL)	1.1 [0.6–3.6]	1.0 [0.5–2.3]	191	0.76	3.0–4.3
TAC (mmol/L)	0.4 [0.08–0.6]	0.5 [0.03–0.6]	146	0.24	>0.35
Cholesterol (mg/dL)	142.5 [115.0–311.0]	125.6 [68.8–293.8]	194	0.37	120–300
HDL (mg/dL)	45.0 [7.7–159.3]	43.0 [8.7–123.1]	228	0.93	^c^
LDL (mg/dL)	88.6 [71.3–162.6]	92.4 [50.5–171.9]	201.5	0.47	^c^
Triglycerides (mg/dL)	56.8 [32.5–88.3]	51.7 [27.7–119.9]	222	0.82	30–200
Total protein (g/dL)	6.3 [4.5–10.5]	6.5 [4.8–9.0]	199.5	0.44	5.4–7.7
Albumin (g/dL)^a^	2.8 [1.9–3.2]	2.5 [1.8–3.6]	150	0.04	2.5–3.6
Amylase (UI/L)	456.2 [252.5–896.7]	570.8 [168.9–1004.0]	149.5	0.56	^c^
Lipase (UI/L)	5.1 [0.9–14.6]	8.0 [1.1–17.6]	149.5	0.49	5.0–200

Mann–Whitney U tests. P values less than 0.05 are considered statistically significant

^a^Significant difference between infected (*n* = 29) and non-infected dogs (*n* = 16)

^b^ *n* = 28

^c^Not available

Significant differences between *Ancylostoma* spp. subclinically infected and non-infected dogs were not observed for other analytes (Table 1).

## Discussion

The main changes observed in female dogs with active shedding of *Ancylostoma* spp. eggs were increases in serum CRP and Hp, UIBC and decreases in iron and IGF-1. Although male dogs also become subclinically infected, only females dogs were chosen for investigation because they are directly involved in contaminating puppies due to the arrested development of *Ancylostoma* spp. larvae. Furthermore the current neutering program at São Paulo State University – Veterinary Teaching Hospital, *campus* of Botucatu, Brazil, is specific for female dogs, which includes controlling the dog population and parasitological diseases and thus enabled sample collection.

C-reactive protein is a major acute phase protein and a sensitive marker of inflammation in dogs and has been used as a biomarker of infectious diseases [15–18]. Haptoglobin is a moderate APP in dogs [16, 19]. The results obtained in our study could indicate a mild inflammatory response that could be associated with the blood-feeding adult stage of the parasite [11], since the CRP median values of subclinically infected dogs were above our reference range (Table 1) (>12 μg/mL) but lower than the major magnitude increase (10 to 100 fold) stimulated by an acute inflammatory response [16].

The low iron and higher UIBC could reflect a situation of functional iron deficiency being compensated for by the mobilization of iron stores producing a higher transportation capacity of iron and thus increases in UIBC [20]. The decrease in iron could be due to inflammation associated with the damage caused by the worms while attached at the small intestine mucosa [21, 22]. It may also be due to its active blood feeding from lacerated capillaries with subsequently lysis of erythrocytes by pore formation and hemoglobin release into the lumen of the parasite’s intestine, resulting in loss of serum proteins, intestinal inflammation and hemorrhage [22, 23]. Ferritin concentrations did not change significantly in our study probably reflecting a mixed effect of increase due to inflammation and decrease due to the mobilization of iron stores to compensate the iron losses [24]. In vivo studies [23, 25] suggested that host iron status mediates *Ancylostoma* spp. pathogenesis, in either parasite development or host susceptibility to infection. Apparently, *Ancylostoma* spp. infection modulates iron metabolism resulting in enhanced absorption from the intestine to compensate parasite associated-blood loss, but it seems that in our study this increased absorption was not enough to avoid a decrease in serum iron in infected dogs [24, 25].

We did not find low hematocrits or changes in mean cell volume or any other sign of iron deficiency in these dogs in CBCs, but the CBCs in our study were made with an impedance counter and reticulocytes indexes were not analyzed. It could be that in infected dogs, despite the lack of changes in CBCs, reticulocyte indexes could reflect changes both related with the situation of iron loss [26] or inflammation [27]. Although ideally reticulocyte counts and indices should have been investigated, these analytes are not typically determined in non-anemic dogs.

The observed decrease in IGF-1 could be due to a variety of causes, one of these could be the inflammation. Insulin-like growth factor – 1 is a negative acute phase protein as its concentrations decreases due to its inhibitory effect of proinflammatory cytokines on the IGF-1 expression in the liver [28]. Moreover, serum IGF-1 concentration decrease in long-term dietary restriction and reduced body size in dogs, and it could be that hookworms can reduce intestinal absorption and produce a decrease in body weight, therefore could also reduce serum concentrations of IGF-1 [29, 30].

It is known that in *Ancylostoma* spp. infection, there can be changes in serum analytes in animals with clinical signs of disease; however, in our study, the dogs did not show clinical signs of parasitism. The changes found in this study could provide an overview of the biochemical effects produced by *Ancylostoma* spp. subclinical infection, which should be considered in differential diagnosis, especially because it is a parasite with zoonotic potential.

No changes were observed in amylase, lipase, and TLI or on the markers related with lipid metabolism (cholesterol, HLD, LDL, butyrylcholinesterase) of the adult female dogs evaluated in this study. This indicates that the subclinical form of infection with this parasite does not affect the pancreas or lipid metabolism. In addition the lack of changes in PON1 and TAC would indicate the absence of an evident oxidative stress in this condition. Although fecal flotation is the most common diagnostic tool in routine examinations and the standard methods were used in this study [31], the major limitations of this study were that a negative result does not mean the dog had had no previous infections with *Ancylostoma* spp., and the anthelmintic administration previous history was not available.

## Conclusions

Changes in markers of inflammation and iron metabolism were detected in subclinically infected dogs with *Ancylostoma* spp. parasites. This study contributes towards an understanding of the host-parasite interaction and to improve the knowledge about the pathology associated with this parasite infection. In addition, *Ancylostoma* spp. infection should be considered in differential diagnosis in apparently healthy dogs showing these changes in APPs and iron metabolism, especially in areas where this parasite is endemic.

## Methods

### Animals

The study population comprised of 45 client-owned adult female mixed breed dogs (1 to 3 years old; 10 to 20Kg), mean body condition score of 3, admitted to the São Paulo State University Veterinary Teaching Hospital of the Faculty of Veterinary Medicine and Animal Science (FVMZ) for elective ovariohysterectomy in the Population Control Program of São Paulo State University - Faculty of Veterinary Medicine and Animal Science. Inclusion criteria included the availability of complete clinical records as part of the routine care and ultrasound evaluation discarding pregnancy. The stage of oestrus cycle was not determined. The dogs included in the study showed no abnormal findings on physical and clinical examination such as cachexia, weight loss or diarrhea, no anemia or leukocytosis or any other considerable change in hemograms. Only serum remaining after other relevant analyses of importance for diagnostic work-up was used in the present study, and this approach was approved by the Faculty’s Animal Experimentation Ethics Committee of the São Paulo State University – FMVZ, UNESP, *campus* Botucatu (protocol number 245/2011-CEUA).

### Laboratory testing

#### Fecal testing

Fecal samples were collected rectally from each dog and underwent floatation [32, 33] using sodium chloride saturated solution and zinc sulphate centrifugal flotation [33, 34]. Fecal eggs determination was done on the same day of sample collection to avoid temperature interference as the samples were not refrigerated or frozen. Both methods were performed on all samples and the results were in agreement [31]. *Ancylostoma* spp. eggs were 55 to 75 μm by 34 to 47 μm [31]. The dogs were divided into 2 groups. Group 1: with negative fecal flotation (no infection). Group 2: with the observation of fecal *Ancylostoma* spp. type eggs/x 40 objective fields (subclinical infection). Group 1 included 16 dogs and group 2 included 29 dogs.

#### Blood analysis

Blood samples were obtained by jugular vein puncture pre-operatively in tubes with EDTA (BD Vacutainer® K2 EDTA Blood Collection Tube; Becton, Dickinson and Company, USA), to perform the hemogram for every animal. Aliquots of the samples (3 mL) were also placed in plain tubes with gel separators (BD Vacutainer® Blood Collection Tube; Becton, Dickinson and Company, USA) were allowed to clot at room temperature, centrifuged (1,500 × g for 5 min) and the harvest sera were stored in Eppendorf microtubes, stored at −20 °C with biochemical analyses performed within six months.

##### Hemograms

A full blood count was performed in every animal using an automated Hematology Analyser (Ebram 18 Hemascreen®). There was no anemia or leukocytosis or any considerable change in hemograms.

##### Biochemistry analysis

C-reactive protein (CRP) concentration was measured using a human immunoturbidimetric assay (CRP OSR 6147 Olympus Life and Material Science Europe GmbH, Lismeehan, O’Callaghan’s Mills, Co., Clare, Ireland), previously used in dogs [35]. Serum haptoglobin (Hp) concentrations were measured via hemoglobin binding assay previously validated for use in dogs [36]. The concentration of IGF-1 and TLI in the serum samples were analyzed by an automated, solid-phase, enzyme-labeled chemiluminescent, immunometric assay (Immulite IGF-1 and TLI assays; Diagnostic Products, Los Angeles, CA, USA) previously used and described in dogs [37, 38].

Serum iron (Iron OSR6186, Beckman Coulter), UIBC (UIBC OSR6124, Beckman Coulter) and ferritin (Tina-quant Ferritin Gen.4, Roche Diagnostic GmbH) concentrations were determined via quantitative assays previously validated [39].

Serum BChE activity was measured using a previously reported method [24] using butyrylthiocholine iodide as substrate and adapted for an automated analyser. By this way the activity of BChE, which represents the total amount of ChE in dog serum, could be quantified [40].

Serum PON-1 was determined with a method previously validated for dogs [41] using p-nitrophenyl acetate as substrate and TAC was measured using a colorimetric method [42].

Total serum HLD, LDL, cholesterol, triglycerides, total protein, albumin, amylase, and lipase were measured using Olympus commercial kits [43, 44].

All assays were measured on an automated biochemistry analyzer (Olympus 600 automatic chemistry analyzer; Olympus Europe GmbH, Hamburg, Germany), with the exception of IGF-1 and TLI that were measured in a chemiluminescent analyser (Immulite, Diagnostic Products, Los Angeles, CA, USA). The biochemical determinations were carried out at the Interdisciplinary Laboratory of Clinical Pathology, INTERLAB – UMU, University of Murcia, Spain.

All assays showed a within run imprecision of less than 10 %.

### Statistical analysis

All statistics were performed using statistical software (GraphPad Version 6 for Windows, GraphPad Software Inc., San Diego, CA, USA). Data are reported as median and range. All variables were first assessed for normality using the Shapiro-Wilk test. Mann–Whitney *U* test was used as all of the data were not normally distributed. Statistical significance was set at *P* < 0.05 for all analyses.
